# Mediation models of anxiety and depression between temperament and drive for thinness and body dissatisfaction in anorexia nervosa

**DOI:** 10.1007/s40519-022-01397-4

**Published:** 2022-04-23

**Authors:** Allan Jérolon, Vittorio Perduca, Nadia Delsedime, Giovanni Abbate-Daga, Enrica Marzola

**Affiliations:** 1 CNRS, MAP5 UMR 8145, Université de Paris, F-75006 Paris, France; 2 Eating Disorders Center, Department of Neuroscience, Rita Levi Montalcini”, Hospital “Città della Salute e Della Scienza”, University of Turin, Via Cherasco 11, 10126 Turin, Italy

**Keywords:** Eating disorders, Eating psychopathology, Personality, Comorbidity, Body image

## Abstract

**Purpose:**

Anorexia nervosa (AN) is a life-threatening condition in which temperament, anxiety, depression, and core AN body-related psychopathology (drive for thinness, DT, and body dissatisfaction, BD) are intertwined. This relationship has not been to date disentangled; therefore, we performed a multiple mediation analysis aiming to quantify the effect of each component.

**Methods:**

An innovative multiple mediation statistical method has been applied to data from 184 inpatients with AN completing: Temperament Evaluation of Memphis, Pisa, Paris, and San Diego Autoquestionnaire, Eating Disorders Inventory-2, State-Trait Anxiety Inventory, and Beck Depression Inventory.

**Results:**

All affective temperaments but the hyperthymic one were involved in the relationship with DT and BD. Only the anxious temperament had a significant unmediated direct effect on DT after the strictest correction for multiple comparisons, while the depressive temperament had a significant direct effect on DT at a less strict significance level. State anxiety was the strongest mediator of the link between affective temperament and core AN body-related psychopathology. Depression showed intermediate results while trait anxiety was not a significant mediator at all.

**Conclusion:**

Affective temperaments had a relevant impact on body-related core components of AN; however, a clear direct effect could be identified only for the anxious and depressive temperaments. Also, state anxiety was the strongest mediator thus entailing interesting implications in clinical practice.

**Level of evidence:**

V, cross-sectional study.

**Supplementary Information:**

The online version contains supplementary material available at 10.1007/s40519-022-01397-4.

## Introduction

Anorexia nervosa (AN) is a severe mental disorder characterized by abnormal patterns of eating behaviors leading to life-threatening emaciation, several organic and psychological sequelae, and high mortality [1]. It is a mental disorder of unknown etiology with typical onset in female adolescents and with a lifetime prevalence in women of up to 2–4% [2, 3]. AN sufferers exhibit an egosyntonic resistance to eating leading to significantly low body weight, intense fear of gaining weight, body image distortions, and severe weight loss due to a relentless pursuit of thinness and restrictive eating [4]. Patients with restricting AN (R-AN) lose weight by dieting and exercising while those with binge-purging AN (BP-AN) also engage in binge eating and/or purging behaviors [4]. The majority of patients (50–70%) improve or recover, 20% of cases develop a protracted course of illness, and 5–10% die [1].

Psychiatric comorbidity is a key-element as a pathogenetic and maintaining factor in AN, also given its impact on body image concerns [5]. Recent studies on patients with AN reported comorbidity rates of 40% for depression and up to 80% for anxiety disorders, with 20–30% for generalized anxiety disorder, and > 50% for social anxiety disorder [6, 7]. Interestingly, data support also the other way round, since patients with major depressive disorder and anxiety disorders are diagnosed more often with a lifetime eating disorder when compared to people without affective or anxiety disorders [8] thus supporting a shared genetic and environmental vulnerability [9, 10]. However, the debate is open on the time-relationship between anxious and depressive symptoms and AN onset and on their role as maintaining factors for AN. For example, the literature supports the onset of anxiety disorders as preceding AN [11–14] with heightened anxiety persisting after recovery from AN [15, 16]. Therefore, these data suggest an underlying anxious trait partially independent of nutrition. Also depression is known to be a key element in AN [17, 18] and, similarly to what happens with anxiety, the onset of depression can precede AN [12, 19] but the time-relationship with AN (e.g., predisposing factor versus consequence of AN-related severe malnutrition) is currently debated [20]. Importantly, anxious comorbidity negatively impacts on AN prognosis [21, 22] and patients with AN and depression tend to be unresponsive to antidepressants [23], to gain less weight in treatment [24, 25] and to report a mood relapse after recovery from the eating disorder [23]. In this vein, a network analysis approach can be of help in overcoming the categorical classification and conceptualization of psychiatric disorders. Using such analyses, anxiety and depression are the nodes with the highest centrality in the constellation of AN-related symptoms [17, 18].

Moreover, research consistently supported the role of temperament as a vulnerability and maintaining factor [26–29] as well as an outcome predictor for AN [30]. Interestingly, these traits – including anxiety and negative emotionality often predate the onset of AN and persist to some extent also after recovery [26, 31–34]. Affective temperaments have been applied to the field of AN [35–37] because helpful in bridging the gap between personality and comorbidity profile in AN. In fact, affective temperaments can be defined as one’s own constitutional, genetically-based, and biologically-determined emotional reactivity to the environmental stimuli that is stable over time [38]. Akiskal and coworkers [39], in the wake of following Kraepelin seminal work on “fundamental states” and Kretschmer's conceptualization of personality, conceptualized an array of affective conditions (depressive, cyclothymic, hyperthymic, irritable, and anxious) which represent a gradient of psychopathological nuances ranging from physiological to clinical conditions. Earlier data from our group provided support to the relevance of the anxious and depressive temperaments in increasing the likelihood of full-blown anxious and depressive comorbidities in patients with AN [36] and specific AN psychopathology, even after controlling for current anxiety and depressive symptoms [37]. These findings mirror those from other fields of psychiatry, including panic disorder [40], bipolar disorders [41], and suicide [42].

However, currently little is known about the role of temperament as compared to that of anxiety and depressive symptoms on core aspects of AN psychopathology (i.e., drive for thinness [DT] and body dissatisfaction [BD]). As stated earlier, it is well-established that anxiety and depression are interwoven with AN [17, 18] but the quantification of their role is to date missing. Statistical mediation analysis, whose goal is to decompose the causal effect a variable has on an outcome into the sum of indirect effects through intermediate variables referred to as mediators and a direct effect, could address well this research question. To date, literature on anxiety and depression as mediators of eating core aspects in AN is in its infancy. In fact, the available studies suggest anxiety as a mediator of the association between appearance-related stress and eating disorder behaviors [43] and between emotional dysregulation and drive for thinness [44]. Still, depression was reported to be a mediator between clinical variables and alexithymia [45]. Importantly, research needs to deepen the potential role of emaciation and age as confounders in this context. In fact, it is well-known that patients’ emaciation can sometimes exaggerate certain patients’ premorbid characteristics as well as their dysphoric state [28]. Less is known on the potential role of age on temperament. However, it should be borne in mind that a study found age as impacting on the cyclothymic temperament [46].

Given the current gaps in literature, with this study, we aimed to investigate in inpatients with severe AN, using an innovative statistical method able to handle multiple and potentially correlated mediators, a mediation models including affective temperaments as predictors, symptoms of anxiety and depression as mediators, and core AN body-related psychopathology (namely DT and BD) as outcomes. DT describes patients’ extreme fear of gaining weight and desire to be thinner while BD indicates the negative subjective evaluation of one’s own body (e.g., stomach, thighs). Given their crucial role in AN psychopathology, both factors represent maintaining factors of AN [47, 48]. With more detail, we hypothesized that marked traits of depressive, cyclothymic, irritable and anxious temperaments would entail greater anxiety and depressive symptoms and more marked DT and BD. The hyperthymic temperament instead would entail a protective effect. Also, we expected to find a positive association between BMI and all variables (i.e., the greater the BMI the greater the depressive symptoms) as reported in literature [49]. It is also noted that as BMI decreases in AN, body dissatisfaction values decrease as measured by the Body Shape Questionnaire and Eating Disorder Examination-Questionnaire [50]. The hypothesis on age was more exploratory but we expected to find a positive association instead (i.e., the greater the age the more marked the other variables). That said, our overarching goal is to shed light on the “quantity” of effect directly underpinning the association between temperament and core aspects of AN versus the extent to which such an association “goes through” patients’ anxiety and depression levels. We expected to find both anxiety and depression symptoms as significant mediators of the relationship between affective temperaments and core AN psychopathology.

## Methods

### Participants

The initial sample was composed of 209 female inpatients consecutively hospitalized at the ward of the Eating Disorders Center of the “Città della Salute e della Scienza” hospital of the University of Turin, Italy. All patients were admitted voluntarily. However, nine patients were excluded from this study because of not meeting the inclusion criteria, 4 candidates failed to provide valid written informed consent, and 12 individuals returned an incomplete assessment. Therefore, we finally enrolled 184 participants with AN.

Upon admission, experienced psychiatrists collected socio-demographical and clinical information, and a trained nurse measured patients’ weight and height to calculate body mass index (BMI). Inclusion criteria for this study were the following: (a) full diagnosis of AN as evaluated by an experienced psychiatrist with the Structured Clinical Interview for DSM-5 [51] upon admission; (b) female gender; (c) no substance dependence; (d) no psychotic –spectrum disorders; (e) no organic comorbidities (e.g., epilepsy).

The Ethical Committee of the Department of Neuroscience of the University of Turin, Italy, approved this study and all patients provided their written informed consent.

### Measures

All participants completed the following assessments within the first week of the hospital stay:

- Temperament Evaluation of Memphis, Pisa, Paris, and San Diego Autoquestionnaire (TEMPS-A). In this study, we used the validated Italian version of the TEMPS-A [52]. The TEMPS-Autoquestionnaire is a 110-item (109-item for men) self-administered, Yes-or-No type questionnaire that allows measuring affective temperamental traits. It assesses depressive (items 1–22), cyclothymic (items 23–42), hyperthymic (items 43–63), irritable (items 64–84), and anxious (item 85–110) temperaments. Cronbach’s alpha of the sample 0.94.
- Eating Disorders Inventory-2 (EDI-2). The Italian version of the EDI-2 [53] is a psychometrically robust (Cronbach’s alpha of the sample 0.97) self-report measure of disordered eating attitudes, behaviors, and personality traits common to individuals who are diagnosed with an eating disorder. Eleven subscales evaluate the symptoms and psychological correlates of eating disorders; high scores reflect more severe pathology.
- State-Trait Anxiety Inventory (STAI). The Italian version of the STAI [54] is a widely used, validated psychometric instrument, that allows distinguishing between two different dimensions of anxiety: the state anxiety (S-anxiety), a transitory state, and trait anxiety (T-anxiety), a stable individual disposition. These two types of anxiety are measured by two different, 20-items, self-report scales. For each item, the severity of anxious symptoms is rated from 1 (not at all/almost never) to 4 (very much so/almost always) and a global score from 20 to 80 is assessed. Cronbach’s alpha of the sample 0.97.
- Beck Depression Inventory (BDI). The BDI [55] is a well-validated instrument used to assess depressive symptomatology severity (Cronbach’s alpha of the sample 0.95). It is composed of 13-items, defining a scale ranging from minimal (scores from 0 to 4) depressive symptoms to severe depression (scores from 16 to 39). Mild (scores from 5 to 7) and moderate (scores from 8 to 15) scores are also available.

### Statistical analysis

Causal mediation analysis comprises statistical methods to decompose the causal effect of an exposure on an outcome into indirect effects through measured intermediate variables called mediators, and a direct effect, while adjusting for confounders [56]. In this context, direct effect comprises both the true causal effect of the exposure on the outcome that is not mediated by any intermediate variables and, possibly, the effect that goes through mediators that are not measured. In this work we adopted the counterfactual framework for mediation analysis. According to this framework, the direct, indirect and total effects are defined as statistical parameters independently from a predefined model and are expressed as differences of expected counterfactual outcomes. As such, these effects are measured in the scale of the outcome. The starting point of any mediation analysis is the assumption of a causal model for the data generation process in the form of a Directed Acyclic Graph (DAG), a diagram where the nodes correspond to the variables of interest and the directed edges represent potential direct causal effects. Such DAGs generally reflect the current knowledge about the phenomenon under investigation. Once a DAG is built and it verifies identification conditions generally known as sequential ignorability, mediation analysis tools estimate direct and indirect effects from the data. In this work, we applied a recently published method that makes it possible to deal with multiple mediators showing residual correlation after adjustment for confounders [57]. This method applies a quasi-Bayesian algorithm to estimate the total causal effect, the direct effect, the joint indirect effect of all mediators and the mediator specific indirect effects from the linear model of the outcome given the exposure, the mediators and the observed confounders and the linear models of the mediators given the exposure and the observed confounders.

**Fig. 1:**
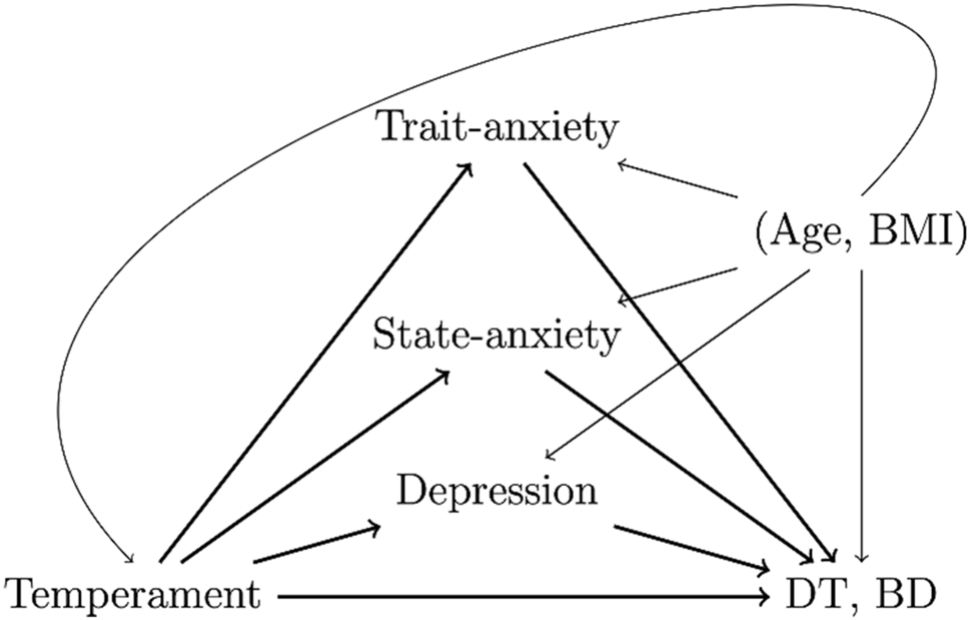
Directed Acyclic Graph (DAG) showing the causal assumption between the variables of interest

In this work, the exposures of interest are affective temperaments (depressive, cyclothymic, hyperthymic, irritable, or anxious), the potential mediators are state and trait anxiety (as measured by the S-STAI and the T-STAI, respectively) and depressive symptoms (as measured by the BDI), and the outcomes are key aspects of AN psychopathology (as measured by DT and BD on the EDI-2). For each affective temperament-eating psychopathology pair we assume the DAG depicted in Fig. 1 and realize a separate mediation analysis. For each of these 10 analyses, we started by fitting (1) the linear model of the eating disorder under consideration given the three potential mediators and the temperament and (2) the linear models of the three potential mediators given the temperament. These models were adjusted for age and BMI. We then applied the R package multimediate^1^ to these two models to estimate the effects of interests together with confidence intervals and p-values. Roughly speaking, causal effect estimates represent the average change of the considered outcome in response to a change of one unit of the temperament while holding constant the mediators (direct effect), of the mediators while holding fixed the temperament (indirect effects). For more precise definitions of the natural direct and indirect effects adopted in this work we refer to [54]. In each mediation analysis, to control the family-wise error rate at the nominal 0.05 value, we adopted the threshold 0.05/6 = 0.0083 for significance calling (intermediate local threshold). Moreover, to control the family-wise error rate in the totality of performed tests, we also considered the threshold 0.05/60 = 0.00083 (global threshold). All analyses were done in R.

## Results

### Clinical characteristics of the sample

Out of the whole sample (*n* = 184), 129 patients (70.1%) were diagnosed with the R-AN and 55 (29.9%) with the BP-AN subtype of AN. Patients were all Caucasian and their mean age was 23.7 ± 8.7 years, mean admission BMI was 14.4 ± 1.8, and mean duration of illness was 6 ± 7.8 years. Sixty patients (39.2%) reported previous AN-related hospitalizations and 102 (66.7%) were on medications upon admission. Concerning comorbidity, 61 patients (40.1%) had a comorbid psychiatric disorder (i.e., a formal diagnosis of major depression or anxiety disorders). Supplementary Table 1 shows further summary statistics about the variables depicted in Fig. 1.

### Mediation analysis of anxiety and depression

Supplementary Table 2 shows the estimates of the coefficients in all the linear models needed for the ten mediation analyses.

**Fig. 2:**
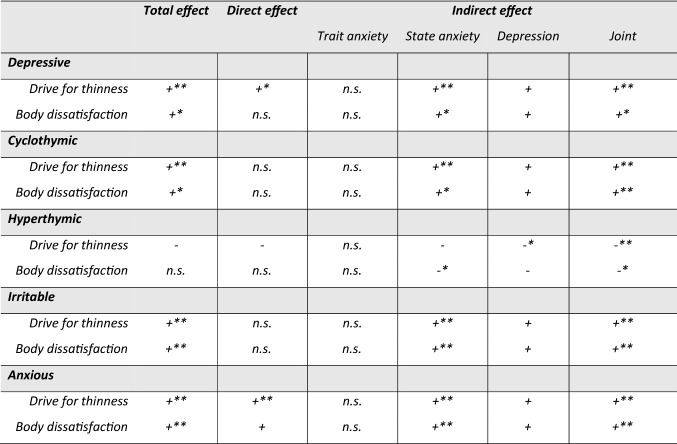
Mediation between affective temperaments, anxiety and depression on drive for thinness and body dissatisfaction at a glance. Legend: + positive estimate (*p* value < 0.05);—negative estimate (*p* value < 0.05); n.s. non-significant; * significant after Bonferroni correction at the intermediate threshold *p* value < 0.05/6 = 0.0083; ** significant after Bonferroni correction at global threshold *p* value < 0.05/60 = 0.00083

**Fig. 3:**
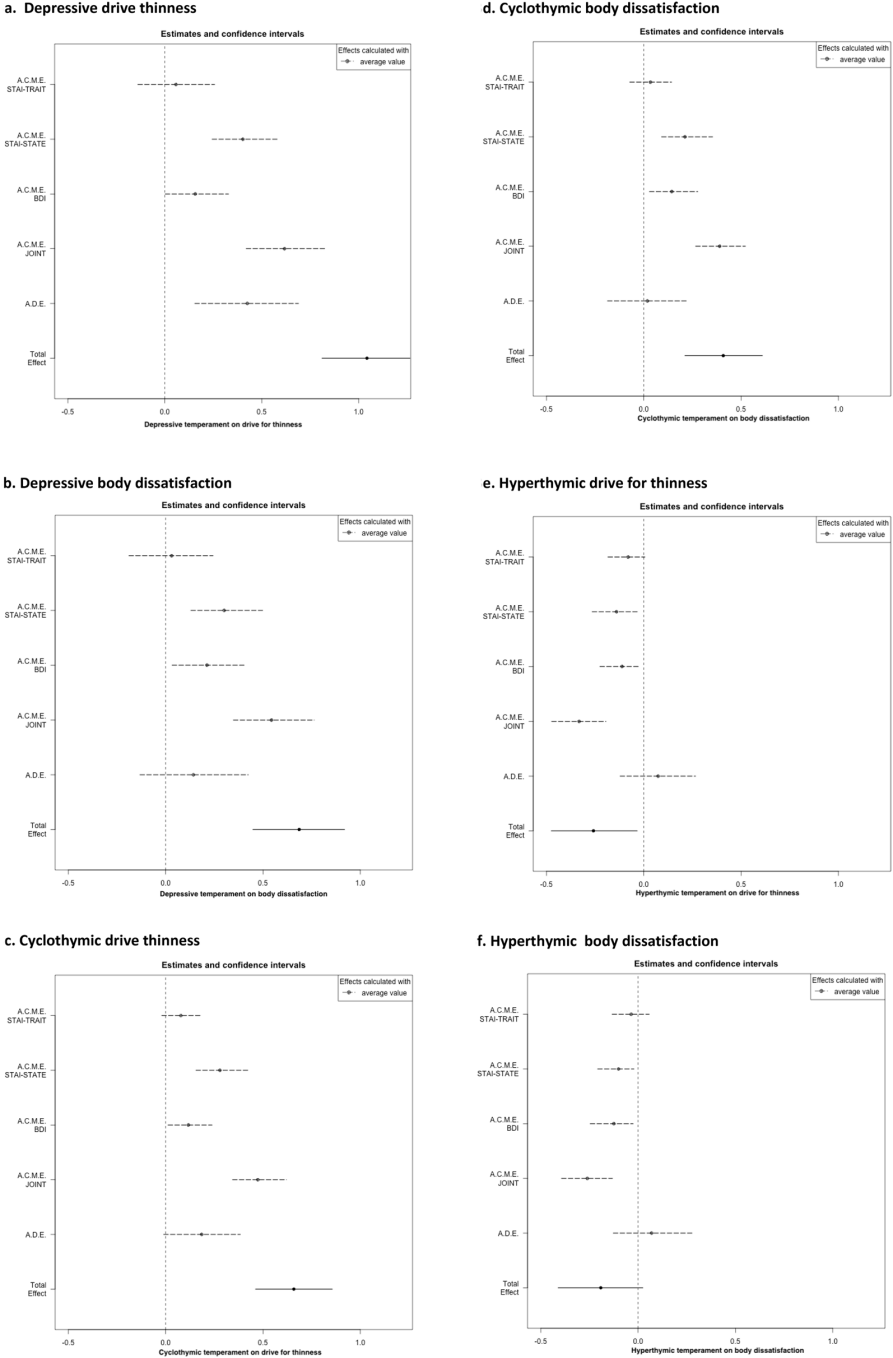

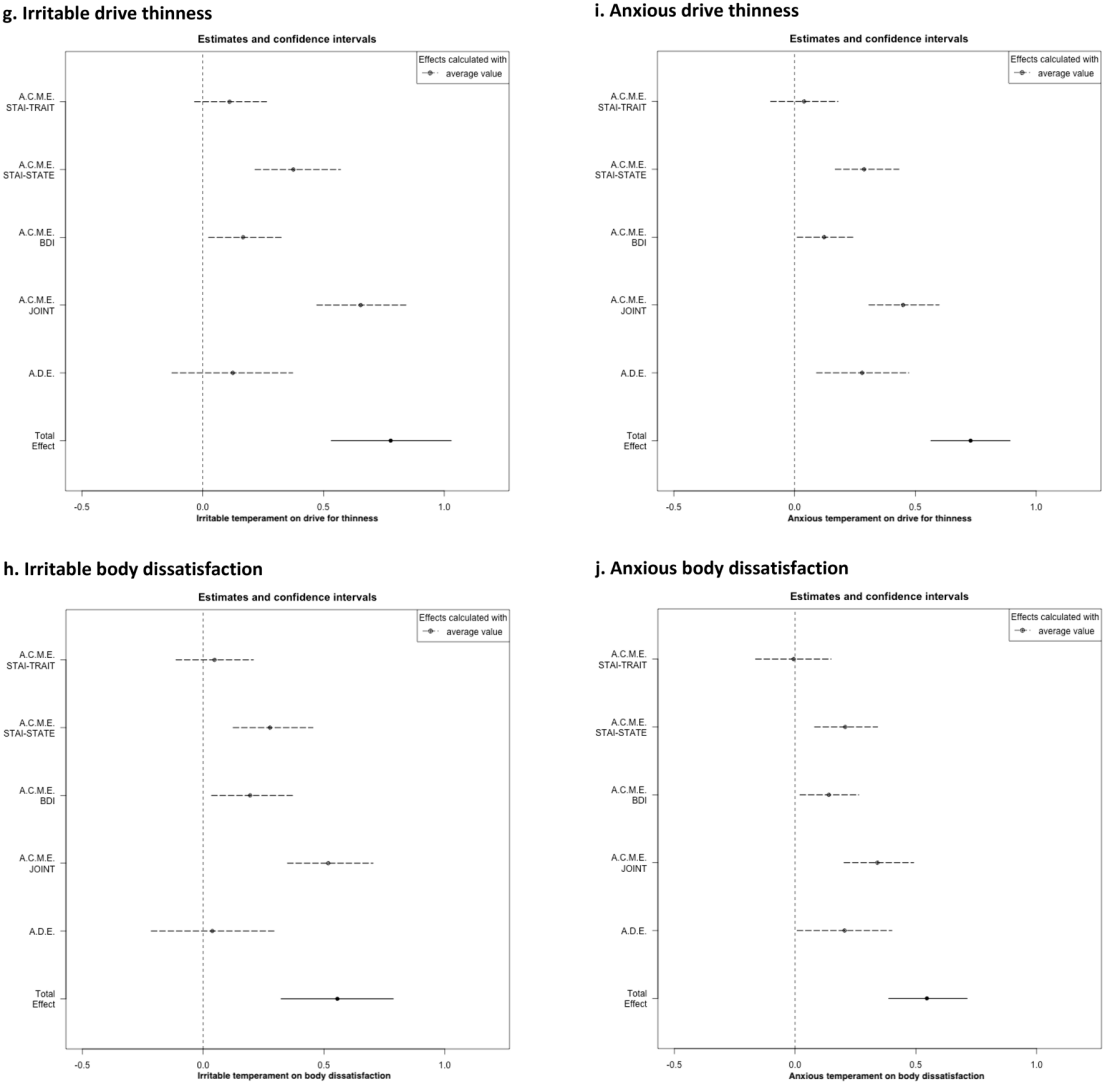
Mediation models of anxiety and depression between affective temperaments and eating psychopathology and Body Mass Index: estimates and 95% confidence intervals for the following causal effects: A.C.M.E.: Average Causal Mediation Effect (i.e., indirect effect) via individual mediators (STAI-TRAIT, STAI-STATE, BDI) or through all the three mediators taken jointly; A.D.E. Average Direct Effect; Total effect

As reported in Table 1 and Figs. 2, 3, all affective temperaments but the hyperthymic one had a significant total effect on DT and BD.

**Table 1:** Mediation models of anxiety and depression between affective temperaments and eating psychopathology and Body Mass Index

	Drive for thinness§			Body dissatisfaction§		
	Estimate	95% CI for estimate	*p*	Estimate	95% CI for estimate	*p*
TEMPS-A						
Depressive						
Total effect	1.04	0.81, 1.26	** < 8.3e-4**^b^	0.68	0.45, 0.91	** < 8.3e-4**^b^
Average causal mediation effect						
STAI-TRAIT	0.06	− 0.14, 0.26	0.565	0.03	− 0.18, 0.24	0.767
STAI-STATE	0.4	0.24, 0.59	** < 8.3e-4**^b^	0.3	0.13, 0.50	** < 8.3e-4**^b^
BDI	0.16	− 0.001, 0.33	**0.048**	0.21	0.03, 0.41	**0.019**
JOINT	0.62	0.42, 0.82	** < 8.3e-4**^b^	0.54	0.35, 0.76	** < 8.3e-4**^b^
Average direct effect	0.43	0.18, 0.69	**0.002**^b^	0.14	− 0.13, 0.42	0.345
Cyclothymic						
Total effect	0.66	0.46, 0.86	** < 8.3e-4**^b^	0.41	0.21, 0.60	** < 0.001**^a^
Average causal mediation effect						
STAI-TRAIT	0.08	− 0.02, 0.19	0.128	0.03	− 0.07, 0.14	0.533
STAI-STATE	0.28	0.16, 0.42	** < 8.3e-4**^b^	0.21	0.09, 0.35	**0.001**^a^
BDI	0.12	0.01, 0.24	**0.032**	0.14	0.03, 0.28	**0.018**
JOINT	0.47	0.34, 0.62	** < 8.3e-4**^b^	0.39	0.27, 0.52	** < 8.3e-4**^b^
Average direct effect	0.18	− 0.01, 0.38	0.069	0.02	− 0.19, 0.22	0.857
Hyperthymic						
Total effect	− 0.25	− 0.47, − 0.03	**0.024**	− 0.19	− 0.04, 0.02	0.088
Average causal mediation effect						
STAI-TRAIT	− 0.08	− 0.18, − 0.006	0.066	− 0.04	− 0.13, 0.06	0.433
STAI-STATE	− 0.14	− 0.26, − 0.03	**0.012**	− 0.1	− 0.21, − 0.02	0.006*
BDI	− 0.11	− 0.23, − 0.02	0.008^a^	− 0.12	− 0.25, − 0.03	**0.011**
JOINT	− 0.33	− 0.47, − 0.19	** < 8.3e-4**^b^	− 0.26	− 0.39, − 0.13	** < 8.3e-4**^b^
Average direct effect	0.07	− 0.12, 0.26	0.454	0.07	− 0.12, 0.27	0.512
Irritable						
Total effect	0.78	0.53, 1.02	** < 8.3e-4**^b^	0.55	0.32,.79	** < 8.3e-4**^b^
Average causal mediation effect						
STAI-TRAIT	0.11	− 0.03, 0.26	0.126	0.04	− 0.11, 0.21	0.568
STAI-STATE	0.37	0.21, 0.57	** < 8.3e-4**^b^	0.27	0.12, 0.45	** < 8.3e-4**^b^
BDI	0.17	0.02, 0.32	**0.018**	0.19	0.03, 0.37	**0.014**
JOINT	0.65	0.47, 0.84	** < 8.3e-4**^b^	0.51	0.35, 0.70	** < 8.3e-4**^b^
Average direct effect	0.12	− 0.12, 0.37	0.319	0.04	− 0.21, 0.29	0.754
Anxious						
Total effect	0.73	0.56, 0.89	** < 8.3e-4**^b^	0.54	0.39, 0.71	** < 8.3e-4**^b^
Average causal mediation effect						
STAI-TRAIT	0.04	− 0.09, 0.18	0.564	− 0.006	− 0.15, 0.14	0.931
STAI-STATE	0.28	0.17, 0.43	** < 8.3e-4**^b^	0.21	0.1, 0.35	** < 8.3e-4**^b^
BDI	0.12	0.01, 0.24	**0.035**	0.14	0.02, 0.26	**0.018**
JOINT	0.45	0.30, 0.60	** < 8.3e-4**^b^	0.34	0.2, 0.48	** < 8.3e-4**^b^
Average direct effect	0.28	0.09, 0.47	** < 8.3e-4**^b^	0.21	0.03, 0.40	**0.04**

^§^Model adjusted for BMI and age

^a^Significant after Bonferroni correction for multiple comparisons to adjust for all multiple comparisons in each individual mediation analysis (intermediate significance threshold = 0.05/6 = 0.0083)

^b^Significant after Bonferroni correction to adjust for all multiple comparisons in the ten mediation analyses (global significance threshold = 0.05/60 = 0.00083)

Concerning the direct (i.e., unmediated) effect, only two affective temperaments, namely depressive and anxious, showed an unmediated impact on DT, even though the direct effect of depressive temperament on DT was no longer significant after the most stringent correction for multiple comparison. The anxious and hyperthymic temperament had also a significant direct impact on BD and DT respectively, but this was no longer true after statistical correction for multiple comparisons.

About the indirect (i.e., mediated) effect, we found a joint (i.e., anxiety + depressive symptoms) significant effect for all mediators and outcomes considered, even though the joint indirect effect of depressive and hyperthymic temperaments on BD were not significant after global multiple comparison correction. With more detail, when analyzing the specific contribution in the mediation model for each factor (i.e., trait and state anxiety and BDI), trait anxiety did not show any significant mediation effect while state anxiety resulted as a significant mediator of DT for all affective temperaments at least at some level of statistical significance. Depressive symptoms showed statistical significance as mediators of the relationship between all affective temperaments and DT and BD before correction for multiple comparisons; only the mediation of BDI between the hyperthymic temperament and DT was significant at the intermediate significance threshold (see Table 1).

## Discussion

With this study, we aimed to investigate and quantify the entrenched relationship occurring between affective temperaments, anxiety, and depression on core aspects of AN body-related psychopathology, namely drive for thinness and body dissatisfaction. To do so, we assumed the causal models with multiple mediators depicted in Fig. 1 and applied a recently published multiple mediation statistical method [57]. Three main findings emerged. First, all affective temperaments but the hyperthymic one were overall involved in the relationship with core aspects of AN. Second, only the anxious and, to a lesser significance level, the depressive temperaments had a significant direct–that is, unmediated by the measured mediators and thus directly influencing outcomes—on DT while only a trend towards significance was found for the relationship between anxious traits and BD. Third, concerning the mediated effect, state anxiety was the strongest mediator of the link between temperament and core AN body-related psychopathology. Depression showed intermediate results, mediating significantly (and negatively) only the relationship between hyperthymic traits and DT, and trait anxiety was not a significant mediator at all. Notably, these are novel data since represent an innovative empirical attempt not only to disentangle but also to quantify the role of anxious and depressive symptoms in the relationship between temperament and clinical characteristics of real-world patients with AN.

Altogether, these findings are in line with earlier data showing the affective temperaments as relevant in the framework of AN [36, 37]; in fact, all temperaments but the hyperthymic one reported a significant total effect on core body-related aspects of AN psychopathology. It should be also borne in mind that hyperthymic traits are more typical of healthy controls and somehow under-represented in AN [36], potentially contributing to explain this finding. In line with earlier data from our group [36], the irritable and the anxious temperaments showed a similar profile but irritable traits should be further studied given their potential connection with the painful dysphoria commonly experienced by AN sufferers [28].

Nevertheless, when considering specifically the direct effect of the affective temperaments on AN core symptomatology, only the anxious and, to a lesser extent, the depressive traits showed a significant direct effect on DT, a crucial aspect of patients’ clinical presentation. Although it should be remembered that these findings refer specifically to the presented models, thus the direct effect could be influenced by temperament via other pathways than the ones (through the mediators) included in our causal model, this represents a novel finding in the field of AN since no mediation analyses on these matters were conducted so far. Interestingly, concerning BD, the anxious temperament showed a trend towards significance while the depressive one did not reach the significance threshold. Taken together, these findings on one hand confirm the role of anxiety and depression in AN but on the other hand, provide the innovative measurement of their direct effect on AN psychopathology as well. In fact, notwithstanding the consolidated role of anxiety and depression in AN [14, 17, 18], findings from our models allow us to disentangle and quantify the role of the anxious and depressive temperament traits independently of current anxious and depressive symptoms. This was particularly true for the effect of anxious and depressive temperaments on DT while BD was found to be not directly influenced by these traits, with the anxious temperament showing only a trend towards significance not surviving statistical correction. These different mediation pathways (different direct effects on DT and BD) is of interest, since–according to this model—DT seems relatively more influenced by a direct effect of anxious and, to a lesser extent, depressive temperament traits than BD, independently of current anxious and depressive symptoms. This is an intriguing finding since BD would seem to be more symptom-dependent than DT. It is noteworthy that, although a genetic vulnerability has been proposed for both DT and BD [58–60], the latter showed to be markedly influenced by socio-cultural processes as well [48] thus potentially explaining the lack of a direct role of temperament in this regard. In fact, BD is so prevalent in Western Countries to be considered as a “normative discontent” [61]; relatedly, cultural influence resulted to strongly predict BD even after controlling for BMI [62] and influential models have been proposed to link socio-culturally mediated factors to body dissatisfaction [63, 64]. All in all, BD is confirmed as a multi-faceted construct potentially influenced by a myriad of factors that future research may want to analyze. Therefore, from a clinical standpoint, DT and BD may require a different approach in treatment given their partially different “response” to patients’ innate temperament.

Moreover, the other side of the previous finding on the direct effect is the measurement of the causal mediation effect of the mediators considered, namely trait and state anxiety, depressive symptoms, and their joint effect. When considering every single mediator, state anxiety reported the strongest effect (with estimates twice as high as those of depression), depressive symptoms showed intermediate results while trait anxiety did not show any mediation effect. However, their joint mediation effect was greater than that of any mediator as a stand-alone entity. Altogether, our findings show that these three major factors (i.e., temperament, anxiety/depression, and body-related symptoms of AN) are interwoven and should be considered as key elements in the development and maintenance of AN. On one hand, this finding provides useful parallelism to studies of network analysis showing that depressive and anxious symptoms are central in AN psychopathology [17, 18]. Nevertheless, this is the first time that the effect of these highly inter-correlated constructs [24, 65] is estimated. Also, the proposed models provided a scientific measure of an every-day clinical notion, since anxiety, both singularly and jointly measured, was found to significantly mediate psychopathological variables. Furthermore, anxiety has been linked to clinical severity as measured by BMI [66]. Still, in the framework of the intriguing scientific debate on anxiety in AN [9, 14, 33], our findings support earlier literature suggesting severe malnutrition as exacerbating the eating core psychopathology aspects of patients with AN [28] in a fine-grained fashion. If the role of state anxiety was supported, trait anxiety, namely patients’ usual and stable levels of anxiety, did not result to be a significant mediator. This is an unexpected finding, given the relevance of anxiety in AN [15, 21, 67]. On one hand, it could be proposed that trait anxiety shows relevant “shared” and somehow overlapping characteristics with the anxious temperament (at least with respect to how the questionnaires address this construct) thus decreasing the chance to be “detected” by the applied statistical method. However, on the other hand, it could be argued instead that, the marked inpatients’ levels of state anxiety take the lion's share in the model, in keeping with the aforementioned hypothesis on the state-dependent aggravation of the anxiety diathesis [28]. The mediation effect of BDI showed intermediate results instead. In fact, after intermediate Bonferroni's correction, depressive symptoms resulted to negatively mediate only the relationship between the hyperthymic temperament and DT. Although the literature on Cognitive Behavioral Therapy had already shown that depression significantly influences weight gain [25, 68], this is a novel finding that tends to dampen earlier findings [17, 18] since, according to our model, it can be surmised that current anxious symptoms impact more on DT and BD than depression in inpatients with severe AN.

Should these data be confirmed, some interesting clinical implications could be proposed. In fact, our data suggest that—when treating AN sufferers in a severe phase of their disorder–close attention should be paid to patients’ constellation of comorbid psychiatric symptoms. Also, it could be raised that hypothesis that, from a clinical standpoint, working on DT and BD could require different treatment approaches, given the different influence of patients’ innate characteristics on DT and BD. In this vein, temperament-based treatments have been authoritatively proposed and are currently being validated [69]. Additionally, therapeutic interventions may want to tackle the more punctiform anxiety and depressive components in treatment as well. For example, although no medications can reverse the core symptoms of AN, pharmacotherapy could be helpful in this regard [70, 71]. Additionally, from a psychotherapy standpoint, the management of anxiety and depression can improve patients’ motivation and quality of life [72].

In closing, prompted by the state-of-the-art supporting the entrenched nature of AN psychopathology and anxious and depressive symptoms, we designed models trying to disentangle this relationship and, importantly, measure the effect of each component involved. We found that temperament per se has a relevant impact on body-related core components of AN, namely DT and BD. However, a clear direct effect could be identified only for the anxious and, to a lesser extent, the depressive temperaments on DT. Also, state anxiety was the strongest mediator as a standalone component when compared to depression and trait-anxiety. In keeping with earlier literature [24], the joint effect of all mediators resulted to be even stronger than state anxiety alone. The proposed models of mediation of anxiety and depression between affective temperaments and body-related psychopathology of AN allowed to quantify direct and indirect effects that were found to be consistent with every-day clinical practice. In fact, clinicians and researchers who work with AN sufferers know well how innate characteristics (i.e., temperament) and current clinical symptoms (i.e., anxiety and depression) can impact on core symptoms of AN. Therefore, if confirmed by further studies, our data contribute to the ongoing scientific debate aiming to shed light on this complex picture also providing some intriguing clinical implications.

### Strength and limits

Overall, this study has some relevant strengths including the innovative statistical method allowing to measure multiple mediators and their joint effect, the recruitment of “real world” patients with severe AN (no insurance/financial barriers exist, according to the Italian National Health System), the pure and full-blown diagnosis of AN, and the aforementioned clinical implications. Nevertheless, some limitations should be acknowledged as well: we recruited only inpatients so this could hamper data generalizability, the sample could be larger, and a cross-sectional design self-report assessments have been used. Even though we applied an advanced method for mediation inference, future studies on larger samples are needed to expand knowledge on its application. It is important to point out that for this study we did not carry out power or sample size calculations as no methods currently exist adapted to our causal multiple mediation setting. Moreover, we reiterate that our quantitative results are valid as long as our assumption of the causal models relating all the variables at stake that is depicted by the DAG in Fig. 1 holds true, with recent advances in the causal inference literature potentially expanding our knowledge in this regard. The statistical method that we applied relies on strong assumptions among which the lack of measured or unmeasured confounders of the relationships between candidate mediators and response variables [54]. Accordingly, we assumed that temperaments do not have an impact on BMI. Even though there is no literature supporting the finding that temperament itself is able to modify, from a causal standpoint, individuals’ BMI, it is important to put forward this possibly controversial assumption. Another hypothesis we made is that candidate mediators do not causally affect each other. Even though this strong assumption can be seen as a limitation, we highlight that we referred to symptoms (of anxiety and depression) rather than to a formal diagnosis of anxiety or depression and, at such a symptom level, it is reasonable to believe that state and trait anxiety and depression can be much causally unrelated. Finally, we assumed the five temperaments as independent. With that being said, if these findings will be confirmed by further studies, these data could be much informative from a clinical perspective.

### What is already known on this subject?

Research consistently supported the role of temperament as a vulnerability and maintaining factor for anorexia nervosa (AN). Also anxiety and depression are key-elements in AN even from an outcome perspective. However, currently little is known about the role of temperament as compared to that of anxiety and depressive symptoms on core aspects of AN psychopathology (i.e., drive for thinness [DT] and body dissatisfaction [BD]).

### What this study adds?

An innovative statistical method allowing to measure multiple mediators and their joint effect was applied to investigate and quantify the relationship between affective temperaments, anxiety, and depression on DT and BD in AN. This study clarified that affective temperaments impacted on body-related core components of AN; a clear direct effect could be identified only for the anxious and depressive temperaments. Also, state anxiety resulted to have the strongest mediator effect.

## Supplementary Information

Below is the link to the electronic supplementary material.

Supplementary file1 (DOCX 19 KB)

## Data Availability

Data available on request due to privacy/ethical restrictions.

## Footnotes

1 https://github.com/AllanJe/multimediate.
