# Supplementary material for: Mediation models of anxiety and depression between temperament and drive for thinness and body dissatisfaction in anorexia nervosa
Source: Eat Weight Disord. 2022 Apr 23;27(7):2569–81. doi: 10.1007/s40519-022-01397-4 (PMC9556361; doi:10.1007/s40519-022-01397-4)
Supplement: Supplementary file 1 — Supplementary file1 (DOCX 19 KB) [file 40519_2022_1397_MOESM1_ESM.docx]

**Supplementary material.**

**Supplementary Table 1. Clinical characteristics of the sample.**

|  |  |  |
| --- | --- | --- |
|  | Mean (SD) | Min - Max |
| Body Mass Index | 14.4 (1.9) | 9.8 – 17.5 |
| TEMPS-A |  |  |
| Depressive | 12.3 (4.1) | 2 - 22 |
| Cyclothymic | 9.5 (4.9) | 0 - 29 |
| Hyperthymic | 7 (4.3) | 0 - 19 |
| Irritable | 6.6 (3.9) | 0 - 16 |
| Anxious | 13.2 (6.7) | 0 - 28 |
| EDI-2 |  |  |
| Drive for Thinness | 12.5 (7.7) | 0 - 21 |
| Body Dissatisfaction | 14.7 (7) | 0 - 27 |
| STAI |  |  |
| State | 54.4 (14.4) | 23 - 79 |
| Trait | 56.9 (14.4) | 0 - 78 |
| BDI | 15.9 (7.8) | 0 - 35 |

**Supplementary Table 2. Estimates and p-values for all the coefficients in the linear models fitted for the mediation analysis.**

| **Analysis** |  | **AGE** | **BMI** | **STAI-TRAIT** | **STAI-STATE** | **BDI** | **Depressive** | **Cyclothymic** | **Hyperthymic** | **Irrittable** | **Anxious** |
| --- | --- | --- | --- | --- | --- | --- | --- | --- | --- | --- | --- |
| Effects of Depressive on DT and BD | STAI-TRAIT | -0.17(0.0775) | 0.52(0.2459) | NA | NA | NA | 2.13(< 2e-16) | NA | NA | NA | NA |
|  | STAI-STATE | -0.28(0.0118) | 0.27(0.5979) | NA | NA | NA | 1.88(8.16e-14) | NA | NA | NA | NA |
|  | BDI | -0.13(0.0273) | 0.57(0.0322) | NA | NA | NA | 1.09(2.78e-16) | NA | NA | NA | NA |
|  | DT | -0.04(0.35456) | 0.54(0.01552) | 0.03(0.56110) | 0.21(2.18e-07) | 0.14(0.06222) | 0.42(0.00172) | NA | NA | NA | NA |
|  | BD | 0.02(0.747168) | 0.55(0.025878) | 0.01(0.758956) | 0.16(3.17e-4) | 0.19(0.022594) | 0.14(0.341234) | NA | NA | NA | NA |
| Effects of Cyclothymic on DT and BD | STAI-TRAIT | 0.01(0.89) | 1.22(0.018591) | NA | NA | NA | NA | 1.13(4.36e-08) | NA | NA | NA |
|  | STAI-STATE | -0.09(0.448) | 0.68(0.20) | NA | NA | NA | NA | 1.30(1.44e-09) | NA | NA | NA |
|  | BDI | -0.02(0.72955) | 0.85(0.00323) | NA | NA | NA | NA | 0.70(1.45e-09) | NA | NA | NA |
|  | DT | -5.9e-3(0.90355) | 0.60(0.00918) | 0.07(0.12261) | 0.21(4.11e-07) | 0.17(0.03421) | NA | 0.19(0.05308) | NA | NA | NA |
|  | BD | 0.03(0.606611) | 0.60(0.018421) | 0.03(0.518557) | 0.16(2.73e-4) | 0.20(0.014526) | NA | 0.01(0.890522) | NA | NA | NA |
| Effects of Hyperthymic on DT and BD | STAI-TRAIT | -0.08(0.469192) | 1.76(7.42e-4) | NA | NA | NA | NA | NA | -0.99(.06e-05) | NA | NA |
|  | STAI-STATE | -0.20(0.1088) | 1.44(0.0113) | NA | NA | NA | NA | NA | -0.62(0.0104) | NA | NA |
|  | BDI | -0.08(0.207) | 1.20(5.51e-05) | NA | NA | NA | NA | NA | -0.56(1.04e-05) | NA | NA |
|  | DT | -0.02(0.73999) | 0.65(4.34e-3) | 0.08(7.35e-2) | 0.22(9.95e-08) | 0.20(0.01079) | NA | NA | 0.07(0.43) | NA | NA |
|  | BD | 0.03(0.606903) | 0.60(0..016849) | 0.04(0.446584) | 0.16(2.57e-4) | 0.22(9.94e-3) | NA | NA | 0.07(0.516321) | NA | NA |
| Effects of Irritable on DT and BD | STAI-TRAIT | -0.005(0.962443) | 1.14(0.021440) | NA | NA | NA | NA | NA | NA | 1.66(4.19e-11) | NA |
|  | STAI-STATE | 0.12(0.290) | 0.70(0.178) | NA | NA | NA | NA | NA | NA | 1.70(1.91e-10) | NA |
|  | BDI | 0.04(0.53181) | 0.85(2.58e-3) | NA | NA | NA | NA | NA | NA | 0.93(5.94e-11) | NA |
|  | DT | -0.01(0.77855) | 0.63(5.91e-3) | 0.07(0.13549) | 0.22(1.78e-07) | 0.18(0.02363) | NA | NA | NA | 0.13(0.30624) | NA |
|  | BD | 0.03(0.604904) | 0.58(0.018248) | 0.03(0.543723) | 0.16(2.69e-4) | 0.20(0.015093) | NA | NA | NA | 0.03(0.799784) | NA |
| Effects of Anxious on DT and BD | STAI-TRAIT | -0.15(0.117) | 0.48(0.282) | NA | NA | NA | NA | NA | NA | NA | 1.53(< 2e-16) |
|  | STAI-STATE | -0.26(0.016) | 0.20(0.694) | NA | NA | NA | NA | NA | NA | NA | 1.39(5.30e-15) |
|  | BDI | -0.11(0.0546) | 0.62(0.0256) | NA | NA | NA | NA | NA | NA | NA | 0.72(6.91e-14) |
|  | DT | -0.04(0.421857) | 0.54(0.017271) | 0.02(0.581527) | 0.21(6.13e-07) | 0.17(0.026976) | NA | NA | NA | NA | 0.28(3.69e-3) |
|  | BD | 0.01(0.847691) | 0.50(0.041252) | -0.005(0.918769) | 0.15(7.45e-4) | 0.19(0.020140) | NA | NA | NA | NA | 0.20(0.04606) |

*How to read this table:* in order to estimate the causal effects in the mediation model of (say) Depressive temperament on Drive for Thinness (DT), four linear models were fitted in the first four rows:

1. DT = -0.04*Age + 0.54*BMI + 0.03*STAI-TRAIT + 0.21*STAI-STATE + 0.14*BDI + 0.42*Depressive + random error
2. STAI-TRAIT = -0.17*Age + 0.52*BMI + 2.13*Depressive + random error
3. STAI-STATE = -0.28*Age + 0.27*BMI + 1.88*Depressive + random error
4. BDI = -0.13*Age + 0.57*BMI + 1.09*Depressive + random error

These models were then given to the multimediate function of the R package multimediate to simulate counterfactual values of the mediators and the outcome that were then averaged to estimate the causal effects.
